# A Preclinical Model to Assess Intestinal Barrier Integrity Using Canine Enteroids and Colonoids

**DOI:** 10.3390/biology14030270

**Published:** 2025-03-06

**Authors:** Megan P. Corbett, Vojtech Gabriel, Vanessa Livania, David Díaz-Regañón, Abigail Ralston, Christopher Zdyrski, Dongjie Liu, Sarah Minkler, Hannah Wickham, Addison Lincoln, Karel Paukner, Todd Atherly, Maria M. Merodio, Dipak Kumar Sahoo, David K. Meyerholz, Karin Allenspach, Jonathan P. Mochel

**Affiliations:** 1 Department of Pathology, College of Veterinary Medicine, University of Georgia, Athens, GA 30602, USA; 2 Department of Biomedical Sciences, Iowa State University, Ames, IA 50011, USAdjliu@iastate.edu (D.L.);; 3 Department of Animal Medicine and Surgery, College of Veterinary Medicine, Complutense University of Madrid, 28040 Madrid, Spain; drdiazreganon@ucm.es; 4 3D Health Solutions Inc., Athens, GA 30602, USAchristopher.zdyrski@3dhealth.solutions (C.Z.); karin.allenspach@uga.edu (K.A.); 5 Department of Pathology, Precision One Health Initiative, College of Veterinary Medicine, University of Georgia, Athens, GA 30602, USA; 6 Center for Experimental Medicine, Institute for Clinical and Experimental Medicine, 14021 Prague, Czech Republic; 7 Department of Veterinary Clinical Sciences, Iowa State University, Ames, IA 50011, USA; mmaria@iastate.edu (M.M.M.); dsahoo@iastate.edu (D.K.S.); 8 Department of Pathology, Carver College of Medicine, University of Iowa, Iowa City, IA 52242, USA; david-meyerholz@uiowa.edu

**Keywords:** transwell, canine, organoid, Caco-2, TEM, MDCK, TEER

## Abstract

While two-dimensional (2D) cell cultures, such as Caco-2 and Madin–Darby canine kidney (MDCK) cells are widely used in a variety of biological models, these two-dimensional in vitro systems present inherent limitations in replicating the complexities of in vivo biology. Recent progress in three-dimensional organoid technology has the potential to address these limitations. In this study, the characteristics of conventional 2D cell culture systems were compared to those of canine intestinal organoids (enteroids, ENT, and colonoids, COL). Light microscopy and transmission electron microscopy were employed to evaluate the microanatomy of ENT, COL, Caco-2, and MDCK cell monolayers, while transepithelial electrical resistance (TEER) values were measured to assess monolayer integrity. The TEER values of canine ENT monolayers more closely approximated reported TEER values for human small intestines compared to Caco-2 and MDCK monolayers. Additionally, canine ENT demonstrated greater monolayer stability than Caco-2 and MDCK cells. Notably, while all systems displayed desmosomes, canine ENT and COL exclusively produced mucus. These findings highlight the potential of the canine organoid system as a more biologically relevant model for in vitro studies, addressing the limitations of conventional 2D cell culture systems.

## Simple Summary

Immortalized cell lines are often used to model biological systems, such as the intestinal epithelium. Compared to immortalized cell lines, which are composed of identical cell clones, organoids derived from adult stem cells may represent a more accurate biological model, since they can differentiate into specialized intestinal epithelial cell types. In this study, we isolated adult stem cells from dog intestinal samples, which can be obtained with minimally invasive methods. These adult intestinal stem cells were grown into three-dimensional organoids, which recapitulate the superficial layers (epithelium) of the original tissue. The intestinal organoids were examined under a microscope to assess their similarity to the original tissue, then cultured and compared to two immortalized cell lines. The organoids had features similar to intestinal tissue, such as mucus production. In the 2D cell culture system, the organoids formed a more consistent layer than conventional cell lines, demonstrating similar integrity to that of the human intestine. These findings suggest that organoid cultures derived from dog intestinal adult stem cells can effectively be utilized in traditional cell culture systems.

## 1. Introduction

The intestinal barrier performs several essential functions to maintain the body’s homeostasis. These processes include facilitating nutrient, electrolyte, and water absorption, preventing pathogens from crossing the epithelial barrier, and modulation of xenobiotic intake [1]. Biological models that use epithelial cell cultures grown in monolayers are often employed for applications such as measuring drug permeability and are considered the industry gold standard [2]. Caco-2 and Madin–Darby canine kidney (MDCK) cell lines are commonly used immortalized cell lines for in vitro intestinal barrier models. The Caco-2 cell line was established in 1977 by Fogh et al. [3] from human colorectal adenocarcinoma cells, and Madin and Darby [4] isolated MDCK cells from canine renal cells in 1958. Traditionally, these cell lines have been used for permeability assays due to the ability to standardize culture methods. However, there are limitations to using these cell lines, as they only provide a rough estimate of passive drug diffusion and active drug transport through relatively inexpensive culture techniques [5].

**Figure 1:**
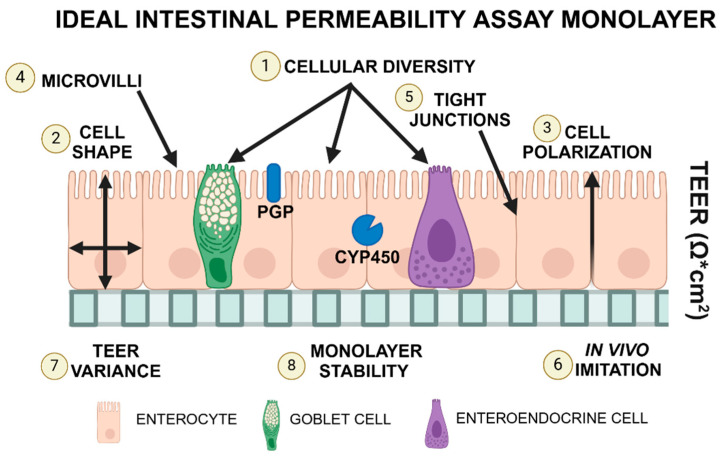
Ideal intestinal permeability assay monolayer. This figure illustrates the desirable properties for a cellular monolayer in a dual-chamber permeable support system, including cellular diversity (1), cell shape (2), polarization (3), the presence of microvilli (4) and tight junctions (5), the ability to imitate in vivo epithelial barrier integrity (6), with stable TEER values (8) and low between-replicates variability (7), and the reliable expression of cytochrome P450 (CYP450), P-glycoprotein (P-gp), and other important transport molecules. Created in BioRender. Corbett, M. (2025) https://BioRender.com/d25v197 (accessed on 7 February 2025).

While these cell lines are readily available and affordable, their ability to accurately predict in vivo results is limited. For example, cytochrome P450 (CYP450), which is responsible for the clearance of orally administered drugs, has variable expression in Caco-2 cells [6]. P-glycoprotein (P-gp), a drug efflux transporter associated with drug resistance, has also been described as highly variable in this cell line [7]. Consistent, physiologically relevant expression levels of these proteins is essential for translating in vitro results to inform potential in vivo studies. The usage of organoid (3D) technology was proposed to overcome the limitations of traditional 2D cell lines. These 3D structures can be derived from several sources, including embryonic stem cells, induced pluripotent stem cells, and Lgr5-positive cells representing an adult stem cell population derived from individual organs [8]. Adult stem cell technology was first developed in 2009 by Sato et al. [9] and is a method of 3D cell culture characterized by self-assembled structures derived from adult stem cells [9]. Adult stem cells can be isolated from organs or tumors of interest using minimally invasive techniques [10]. This results in the formation of organoids that have genetic stability over time without immortalization while maintaining the microanatomical features and functional properties of the original tissue [11]. Canine organoids can form 3D structures that replicate the physiological environment and create a cellular monolayer when placed in a dual-chamber permeable support system [12]. The biological characteristics of an ideal intestinal permeability assay are illustrated in Figure 1. In brief, the cell culture should be capable of representing the full spectrum of differentiated epithelial cells, with comparable formation of cell–cell junctions and epithelial barrier integrity observed in vivo. Epithelial barrier integrity in cell culture monolayers is assessed by measuring transepithelial electrical resistance (TEER) prior to the start of in vitro permeability experiments. In addition to more accurate microanatomy, organoid technology brings an advantage in P-glycoprotein (P-gp)-related studies when compared to traditional Caco-2 culture, as demonstrated by Zhao et al. [13]. Canine adult-stem cell-derived colon organoid 2D monolayers have also been shown to express functional P-gp [14].

For studies focusing on intestinal permeability, the following features (illustrated in Figure 1) are considered desirable in permeability assays:

- Cellular diversity, i.e., the presence of mucin-producing and secretory cells (1); a columnar (2), polarized (3) cellular monolayer and typical microanatomical features of enterocytes such as microvilli (4);
- The presence of tight junctions (5) as evaluated through transmission electron microscopy (TEM) and TEER measurements. TEER values should also replicate those measured in vivo (6), with minimal variations between replicates (7). The focus on TEER values can be justified as this method is the only quantifiable variable obtained in real-time during cell culture.
- Cell monolayers must be stable (8) for several days to allow for permeability assay experiments. Stability for these experiments was defined as a <10% decrease in TEER values in 2 days following the plateau state.

The objective of this study was to characterize canine intestinal organoids grown in a 2D monolayer system using the above criteria and compare them to two immortalized cell lines conventionally used for in vitro permeability assays.

Cell shape is crucial for intestinal permeability, reflecting the evolution from an undifferentiated globoid shape to the traditional columnar enterocyte shape seen in differentiated intestinal epithelia. Height measurements of the cells were used to assess the morphology of enterocytes as described above. TEER value measurements were included to investigate cell monolayer stability and integrity [12]. TEER values were evaluated compared to previously published values obtained employing the Ussing chamber on human intestinal tissue (duodenum, 50–100 Ω*cm^2^; large intestine, 300–400 Ω*cm^2^) [15,16]. Transmission electron microscopy (TEM) was used to evaluate cell ultrastructure and cell-to-cell adhesion.

## 2. Materials and Methods

### 2.1. Canine 3D Enteroid/Colonoid Culture and Monolayer

All protocols were approved by the Iowa State University Institutional Animal Care and Use Committee (Ref. IACUC-18-065).

Eleven healthy adult mix-breed canines (eight neutered males, three spayed females, median age 2.5 years with a range of 5.0 years) were sampled, producing fifteen organoid cell lines that were derived from various sections of the intestines (three from the duodenum, five from the ileum, and seven from the colon). The canine colony was housed at the College of Veterinary Medicine of Iowa State University and operated by Jonathan P. Mochel and Karin Allenspach. Intestinal tissues were harvested through endoscopic, laparoscopic, surgical wedge biopsies, or necropsies. Using other donors, necropsy samples consisted of an additional 12 juvenile donor tissues (7 intact males and 5 intact females) that were harvested after euthanasia for unrelated reasons. These tissue samples produced 12 juvenile organoid cell lines (4 from the duodenum, 3 from the ileum, and 5 from the colon). The median age of this group was 30 days (range = 59 days).

The development and maintenance of canine intestinal organoid cultures for this study followed the standardized methodology established by our group in 2022 [17], with some minor adjustments described below. In summary, 3D canine intestinal organoids from the duodenum, ileum, and colon were isolated and cultured from adult stem cells. The tissues were washed, and the cells were dissociated. The EDTA incubation time was 60 min, and the isolated crypt cells from the supernatant were then co-plated with a subset of the original finely minced tissue. The cell cultures were subsequently passaged enzymatically and expanded at least twice before being cryopreserved in liquid nitrogen. Following thawing, the organoids were cultured from the cryopreserved samples and then passaged twice in a 24-well plate to expand the culture for experimental purposes. The expansion medium was replaced with differentiation medium for the last 5 days of 3D plate growth.

Organoids were prepared for seeding on a dual-chamber permeable support system (Transwell; Corning, Ref. 3470, Corning, NY, USA) following procedures previously standardized by Gabriel et al. [12]. Organoid medium was removed from the plate, and 500 µL of cold Cell Recovery Solution (Corning, Ref. 354253) was added to each well for Matrigel^®^ Matrix (Corning, Ref. 356231) dissolution. The plate was incubated at 4 °C for 30 min. The suspension was then collected in 15 mL tubes and centrifugated at 750× *g* at 4 °C for 5 min. The supernatant was removed with 500 µL of solution left in the tube. Then, 1 mL of TrypLE™ Express (Gibco, Ref. 12604-021, Waltham, MA, USA) was added and incubated at 37 °C in a water bath for 5 min. The tube was subsequently mixed by flicking 10 times and incubated for an additional 5 min in the water bath. Then, 7 mL of Advanced DMEM/F12 (Gibco; Ref. 12634-010) was added to the culture medium to stop the dissociation. The samples were centrifuged at 750× *g* at 4 °C for 5 min, and the supernatant was removed. Trypsanized cell clusters were suspended in 100 µL of the organoid medium and ROCK inhibitor (10 µM) (Y-27632; EMD Millipore Corp.; Ref: SCM075, Burlington, MA, USA) suspension per one transwell insert.

The cell clusters were gently pipetted up and down to release the cells from larger clusters, and the suspension was filtered through a 40 µm cell strainer (Fisherbrand; Ref. 223635547, Waltham, MA, USA) to remove larger clumps. The viable single cells were counted to seed the cells in a density of ~75,000 cells per 1 cm^2^ of the transwell. The transwells were pretreated with 1% Matrigel^®^ Matrix, 1% Collagen I, Rat Tail 3 mg/mL (Gibco, Ref. A10483-01, Waltham, MA, USA) in organoid medium and ROCK inhibitor solution. Each insert was coated with 100 µL of this solution and incubated for 1 h at 37 °C. Afterward, the excess pretreatment solution was carefully removed from each insert.

The desired concentration of cells was seeded in the transwell inserts and gently swirled around in the hood for a stable dispersion of single cells. A total of 700 µL of organoid medium with ROCK inhibitor was added to the wells and incubated for 24 h. After 24 h, the apical medium containing non-adherent cells was removed from the transwells, and 200 µL of medium and ROCK inhibitor solution was added to the transwell. Organoids were cultivated on transwells for 13 days. The culture medium in transwells was changed every alternative day except during weekends. Transepithelial electrical resistance (TEER) values were measured with Millicell^®^ ERS-2 (Millipore, Burlington, MA, USA) every other day until the values reached a plateau phase. The integrity of the monolayer was assessed visually using an inverted microscope (Leica Dmi1, Wetzlar, Germany).

### 2.2. 2D Cell Culture

One cryovial of MDCK cells (ATCC) was transferred from liquid nitrogen to a 37 °C heat bath for two minutes to thaw. After thawing, cells were transferred to a centrifuge tube with 6 mL cold EMEM (ATCC; 30-2003, Manassas, VA, USA). The cells were centrifuged at 100× *g* for five minutes at 4 °C and returned to a sterile hood where the supernatant was removed down to 1 mL. The cell pellet was gently resuspended by pipette mixing and transferred evenly to two 25 cm^2^ flasks with pre-warmed EMEM + 10% Fetal Bovine Serum (Corning; 35-010-CV). Flasks were monitored daily to record the confluence of the cell monolayer. The culture medium was refreshed every 2–3 days by gently pooling and aspirating the old medium with a pipette and replacing it with new, pre-warmed EMEM + 10% FBS to discard detached or non-viable shed cells. When the cell monolayer reached ~80% confluence, the cells were passaged and seeded at 6.67 × 10^3^ cells/cm^2^.

The culture medium was discarded from the flask, and 4 mL of 0.25% Trypsin-EDTA (Gibco; 25200-056) was added to rinse the remaining media from the flask by gently rocking the solution for 5–10 s, removing with a pipette, and then repeating with another 4 mL of trypsin. After rinsing, a final 4 mL of trypsin was added and incubated at 37 °C for 10–12 min, checking every ~4 min under a microscope set at 10× phase contrast to monitor cells as they detach. Once at least 90% of cells were detached and free-floating in the solution, the flask was returned to the hood, and trypsin was deactivated by adding 10 mL of cold EMEM + 10% FBS. The suspended cells were passed through a pre-wetted 40 μm cell strainer into a centrifuge tube and centrifuged at 100× *g* for five minutes at 4 °C. The supernatant was discarded, and the cell pellet was resuspended in 1 mL of media. Viable cells were counted under a microscope using a Trypan Blue staining (Sigma; T8154-100ML, St. Louis, MO, USA) with a hemocytometer, and the sample was diluted to the desired concentration of 2.5 × 10^5^ cells/mL. Once diluted, 100 μL of suspended cells was seeded in each transwell insert, and the plate was gently rotated and checked under a microscope to ensure an even distribution of cells. Finally, 700 μL of warm culture medium was added to the basal well, and the transwell plate was incubated at 37 °C. After 24 h, the apical medium was gently aspirated and replaced with 200 μL of warm culture medium. The medium was refreshed every 2–3 days, and TEER values were recorded every day after initial seeding.

One cryovial of Caco-2 cells (ATCC) was transferred from liquid nitrogen to a 37 °C heat bath for two minutes to thaw. Immediately after thawing, cells were transferred to a centrifuge tube with 6 mL of cold DMEM + 10%FBS (Gibco; 12634-010, Corning; 35-010-CV). Cells were centrifuged at 100 g for five minutes at 4 °C and returned to a sterile hood where the supernatant was removed down to 1 mL. The cells were resuspended by gentle pipette mixing and transferred to one 75 cm^2^ flask. The culture medium was changed every 2–3 days by gently pooling and aspirating the old medium with a pipette and replacing it with new, pre-warmed culture medium to discard non-adherent cells. When cells reached approximately 80% confluence and formed large cell islands, they were passaged and seeded at 1.0 × 10^4^ cells/cm^2^.

The culture medium was discarded from the flask, and 4 mL of 0.25% Trypsin-EDTA (Gibco; 25200-056) was added to rinse the remaining media from the flask by gently rocking the solution for 5–10 s and removing with a pipette. After rinsing, another 4 mL of trypsin was added and incubated at 37 °C for 10–12 min until cell detachment was observed. When at least 90% of cells were detached and free-floating in the solution, the flask was returned to the hood, and trypsin was deactivated by adding 10 mL of cold DMEM + 10%FBS. The suspended cells were passed through a pre-wetted 40 μm cell strainer into a centrifuge tube and centrifuged at 100× *g* for five minutes at 4 °C. The supernatant was discarded, and the cell pellet was resuspended in 1 mL of medium. Viable cells were counted under a microscope using a Trypan Blue staining with a hemocytometer (Fisher Scientific, Waltham, MA, USA), and the sample was diluted to the desired concentration of 2.5 × 10^5^ cells/mL. Once diluted, 25,000 cells (100 μL of suspended cells) were seeded to each transwell, and the plate was gently rotated and checked under a microscope to ensure an even distribution of cells. After seeding the apical insert, 700 μL of warm culture medium was added to the basal well, and the transwell plate was incubated at 37 °C. After 24 h, the apical medium was gently aspirated and replaced with 200 μL of warm culture medium. The medium was refreshed every 2–3 days, and TEER values were recorded every day after initial seeding for two weeks.

### 2.3. Histochemical Staining

All four cell culture models were harvested for histological processing at approximately the two-week mark on the transwell system. Organoids were fixed, processed, and stained as previously described [17]. In short, organoids were submerged in an FAA solution (Formalin–Acetic Acid–Alcohol) for 24 h and then changed to 70% ethanol. Samples were paraffin-embedded, sectioned (4 µm) onto slides, and deparaffinized/hydrated for staining with hematoxylin and eosin (H&E) and Alcian Blue pH 2.5 (Iowa State University Veterinary Diagnostic Laboratory, Ames, IA, USA).

### 2.4. Cell Measurement

**Figure 2:**
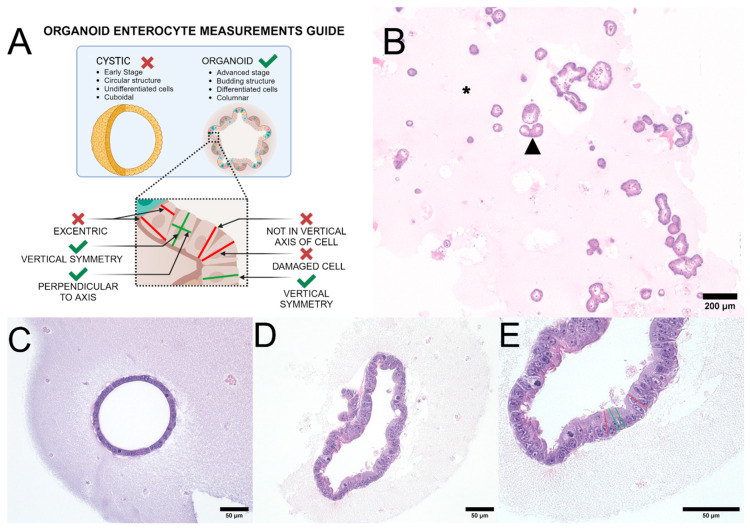
Organoid enterocyte height measurement guide. This figure represents the basic rules followed during the study on canine organoid measurements to ensure accurate results. (**A**) is a schematic that illustrates the structural differences between early cystic organoids and late budding organoids, with examples of correct and incorrect enterocyte height measurements in intestinal organoids (ENT). (**B**) is a representative canine colon organoid (COL) slide, composed of multiple organoids in varying states of differentiation, embedded in extracellular matrix. A black arrowhead indicates an individual COL. The pink material (asterisk) around the organoids is Matrigel, an extracellular membrane matrix: original objective 4×, H&E stain. (**C**) is a representative example of an undifferentiated cystic duodenal organoid, which were excluded from analysis: original objective 20×, H&E stain. (**D**) is an acceptable differentiated budding duodenal organoid. Organoids are budding structures of columnar epithelium morphologically similar to the mucosa of the tissue of origin (in this case duodenum): original objective 20×, H&E stain. (**E**) shows the methods used to measure the 3D organoid cell height at the original objective of 40× (H&E stain). Green lines represent acceptable measurements, while red lines represent cells that were excluded from the analysis as they were not cut along the medial axis. Images were captured using an ECHO Revolution microscope (San Diego, CA, USA). Created in BioRender. Corbett, M. (2025) https://BioRender.com/j00a064 (accessed on 7 February 2025).

Height measurements were performed on H&E-stained slides in a blinded fashion [18]. The minimum number of cells required for inclusion in the cohort was set at 100 measurable cells. The guidelines for measuring height are shown in Figure 2A. Cell height of Caco-2 and MDKC cells on transwells was measured using the same methods as organoids.

The canine organoid culture included two types: (1) cystic organoids (at Day 3–5 of culture) and (2) budding organoids (Day 5–7 of culture) (Figure 2B). Cystic organoids, which mainly consist of stem cells in the early developmental stage, were not measured at this stage as most cells have not yet undergone terminal differentiation (Figure 2C). On the other hand, budding organoids, which mainly consist of mature intestinal epithelial cells, were measured for this experiment (Figure 2D). Any organoids that were damaged after microtome cutting or during the staining process were excluded from the analysis. Additionally, organoids containing cells with smaller nuclei than neighboring cells were also excluded from the cohort to prevent any sectioning plane artifacts or biases (Figure 2E). Epithelial cells were measured from digital images taken at a 40x objective magnification using an Olympus BX43 microscope (Olympus Corporation, Tokyo, Japan). Height measurement parameters were determined in typical budding organoid structures. The height was measured at mid-cell from the apical membrane (at the level of microvilli) to the basal membrane (Figure 2E). Measurements were performed using ImageJ v. 1.53 (National Institute of Health, Bethesda, MA, USA) [19].

### 2.5. Transmission Electron Microscopy (TEM)

Sample processing and imaging methods for TEM were performed according to previously described methods [20]. All four cell culture models were harvested for TEM at approximately the two-week mark on the transwell system.

## 3. Statistical Analysis

Forty-four canine organoid lines were evaluated. The cell height for each group of organoids (adult duodenum *n* = 290 cells; juvenile duodenum *n* = 400 cells; adult ileum *n* = 459; juvenile ileum *n* = 300 cells; adult colon *n* = 687 cells; juvenile colon *n* = 493 cells) and MDCK (*n* = 99) and Caco-2 (*n* = 99) cells was tested for normality using a D’Agostino and Pearson test. All groups except for the MDCK cells were not normally distributed. No outliers were identified using a ROUT method. The cell heights of all groups were compared using a Kruskal–Wallis test with a Benjamini, Krieger, and Yekutieli correction for multiple comparisons (false discovery rate Q = 0.05).

Transepithelial electrical resistance (TEER) values were measured every other day for duodenal organoid monolayers, and every day for MDCK cells and Caco-2 cells. Means, standard deviation, and coefficient of variance (CV) were calculated from three replicate wells per culture type.

All analysis was performed using GraphPad v 10.3.1.

## 4. Results

### 4.1. Canine Organoid Enterocyte Measurements

A Kruskal–Wallis test revealed statistically significant differences in cell height across the eight groups (*p* < 0.001). Measurements of the median height of organoid enterocytes derived from different parts of the intestine and different groups (adult vs. juvenile) are summarized in Table 1, with visual representation in Supplemental Figure S1. Key findings were that organoid cell height differed by intestinal segment in both adult and juvenile derived organoids, adult dog derived organoid cells were taller than juvenile dog derived organoid cells, and MDCK cells grown on transwell inserts were shorter than all other cells. Mean enterocyte height of the adult stem cell-derived organoids from healthy adult dogs differed between intestinal segments: duodenum (14.9 ± 5 µm) vs. ileum (18.6 ± 5.8 µm), *p* < 0.001; duodenum vs. colon (20.55 ± 5.0 µm), *p* < 0.001; and ileum vs. colon, *p* < 0.001. Mean enterocyte height was increased in adult-derived organoid cultures compared to juvenile-derived cultures (duodenum: adult vs. juvenile, 14.9 ± 4.9 µm vs. 10.9 ± 3.0 µm, *p* < 0.001; ileum: adult vs. juvenile, 18.6 ± 5.8 µm vs. 14.0 ± 4.4 µm, *p* < 0.001; colon: adult vs. juvenile, 20.55 ± 5.0 µm vs. 18.3 ± 4.4 µm, *p* < 0.001). MDCK cells were shorter than all other cell types, including Caco-2 cells (*p* < 0.001). Caco-2 cells were shorter than adult ileum organoids and both adult and juvenile colon organoids (*p* < 0.001). Caco-2 cells were the same height as adult duodenum organoids and juvenile ileum organoids (*p* > 0.05), and taller than juvenile duodenum organoids (*p* < 0.001).

### 4.2. Cell Morphology

**Figure 3:**
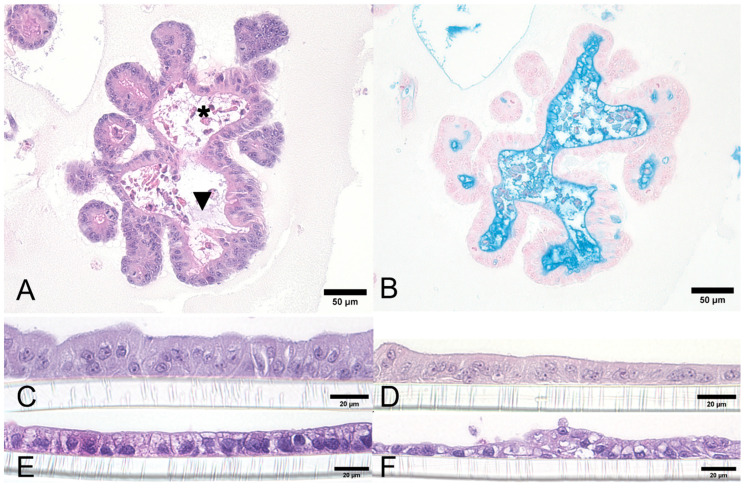
Organoid, Caco-2, and MDCK cell morphology. The morphology of canine intestinal organoids (**A**,**B**). Representative canine COL in 3D culture with mucin production, characterized by wispy, basophilic, intraluminal material ((**A**), black arrowhead). The COL lumen with sloughed cellular debris is indicated by an asterisk; original objective 20×, H&E. Representative canine COL in 3D culture with intraluminal mucin highlighted in bright blue with an Alcian Blue pH 2.5 histochemical stain (**B**); original objective 20×. Comparison of 2D transwell-grown monolayers (**C**–**F**) between a representative canine duodenal ENT monolayer, a representative COL monolayer, and conventional 2D cell lines (Caco-2 and MDCK). Strictly columnar shapes can be observed in the duodenum ENT monolayer (**C**) and columnar to tall cuboidal shapes in the COL monolayer, while more cuboidal shapes are present in the Caco-2 cells (**E**), and polygonal shapes creating a flattened to piling arrangement are present in the MDCK cells (**F**); original objective 40×, H&E. Images were captured using an ECHO Revolution microscope. Created in BioRender. Corbett, M. (2025) https://BioRender.com/n03j283 (accessed on 7 February 2025).

ENT and COL in 3D culture frequently had luminal mucin production (Figure 3A), which was demonstrated with an Alcian Blue pH 2.5 histochemical stain (Figure 3B). The cell shapes after transwell plating were columnar for canine ENT (Figure 3C), columnar to tall cuboidal for canine COL (Figure 3D), cuboidal for Caco-2 cells (Figure 3E), and polygonal to flattened for MDCK cells (Figure 3F).

### 4.3. Transepithelial Electrical Resistance (TEER) Measurements

**Figure 4:**
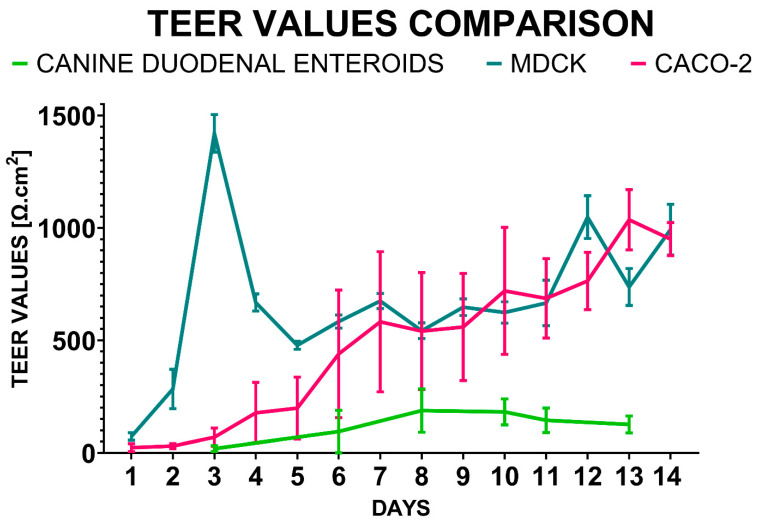
TEER values comparison. The TEER value time-course was observed over a period of two weeks as TEER values were measured for monolayer cultures (mean TEER +/− SD) in a dual-chamber permeable system derived from canine duodenal ENTs, MDCK, and Caco-2 cells.

TEER values measured in the dual-chamber system using a volt/ohmmeter were compared in MDCK, Caco-2, and canine duodenal ENT monolayer cultures (Figure 4).

MDCK TEER values demonstrated relatively low between-replicate variability (CV% = 48.4%), with a peak value on Day 3 (1420 ± 84 Ω*cm^2^, *n* = 12). The TEER values decreased by 65% to 478 ± 18 Ω*cm^2^ on Day 5. By Day 8, TEER values had reached 543 ± 35 Ω*cm^2^ and continued to rise slowly until Day 11 (666 ± 34 Ω*cm^2^).

The Caco-2 TEER values did not exhibit an initial peak but rather consistently increased for 14 days throughout the experiment. However, there was a relatively high variance observed (CV = 69.7%). On Day 10, the TEER measurements showed a value of 720 ± 283 Ω*cm^2^. The highest TEER values for the Caco-2 cultures were observed on Day 13 after seeding (1036 ± 134 Ω*cm^2^).

During the 13 days of culture, the canine duodenal organoid-derived monolayer TEER values increased more slowly with a lower variance (CV = 49.8%), similar to the MDCK cultures. The ENT monolayers in the dual-chamber system reached the plateau phase on Day 10 (182 ± 58 Ω*cm^2^, *n* = 79). The peak TEER values for the canine duodenal ENT monolayer were measured on Day 8, with an average of 188 ± 96 Ω*cm^2^.

### 4.4. Transmission Electron Microscopy (TEM)

**Figure 5:**
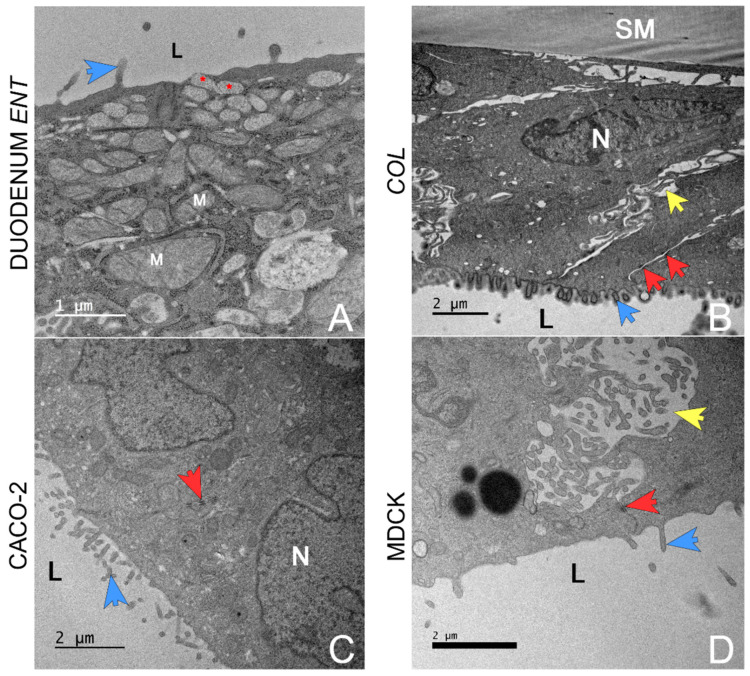
Comparison of 3D organoids plated on a 2D transwell system with 2D cell cultures on transwell systems via TEM. These are the representative transmission electron microscopy (TEM) images. All cultures were imaged on a 2D transwell membrane. (**A**)—Duodenal organoid monolayer with small vacuoles releasing mucin-like material at the apical surface (red asterisk) near the lumen (**L**). Mitochondria are indicated by (**M**). The blue arrow indicates apical microvilli. (**B**)—Colon organoid monolayer displaying polarization; the basolateral border was defined by a permeable support membrane (**SM**) and the apical border by the organoid lumen (**L**). Microvilli (blue arrow) project from the apical membrane into the lumen. The columnar cells contain a centrally to basolaterally located nucleus (**N**). Cells are separated by paracellular spaces (yellow arrow) anchored by desmosomes (red arrow). (**C**)—Caco-2 cells. Note the nucleus (**N**), brush border microvilli (blue arrow) on the luminal surface (**L**), and desmosomes (red arrow). (**D**)—MDCK cells. The apical membrane brush border has rare microvilli (blue arrow) near the lumen (**L**). Note the wide paracellular spaces (yellow arrow) and desmosome (red arrow). Created in BioRender. Corbett, M. (2025) https://BioRender.com/e01p590 (accessed on 7 February 2025).

TEM images were captured from monolayers prepared from canine organoid cells (duodenum and colon), Caco-2 cells, and MDCK cells (Figure 5). The duodenal ENT monolayer was composed of columnar cells growing in monolayers on the permeable support membrane with sporadic overgrowth into two layers. Several cells were observed producing vacuoles with mucin-like contents (Figure 5A), likely representing goblet cell differentiation. Each cell featured microvilli at the apical membrane and exhibited strong attachment to the permeable support with no visible tears in the membrane. The colon organoids displayed similar features, such as polarization, desmosomes, and microvilli brush border formation on the apical membrane; however, no mucus vacuoles were observed (Figure 5B).

Caco-2 cells (Figure 5C) were cuboidal, with stronger overgrowth visible in certain regions of the monolayer creating up to five cell layers on top of each other. Tight junctions and intercellular spaces appeared smaller than in the organoid model. MDCK cells expressed a polygonal shape, and it was difficult to determine their cell borders. The MDCK cells had larger intercellular spaces (Figure 5D). Overgrowth into several layers of cells was present in over 50% of the area of the membrane. MDCK cells had fewer microvilli (Figure 5D).

## 5. Discussion

In this study, canine ENT and COL were compared to MDCK and Caco-2 conventional cell cultures. Based on microscopic morphology and TEER as a measurement of monolayer stability, canine ENT and COL more accurately recapitulated in vivo intestinal tissue compared to the immortalized cell lines, suggesting a potential advantage of using canine intestinal organoid cultures in 2D biological model systems.

Both adult and juvenile canine organoid enterocyte cell heights were higher than MDCK cell heights, more accurately recapitulating the in vivo appearance of intestinal epithelium. Adult canine organoid enterocyte cell heights were higher than both MDCK and Caco-2 cell heights, with the exception of adult duodenal organoids which were the same height as Caco-2 cells. In 2D culture, the tall cuboidal to columnar morphology of canine organoid enterocytes appeared maintained and were subjectively higher than MDCK cells. MDCK cells had a polygonal to flattened phenotype observed both with light microscopy and TEM. Adult canine intestinal organoid cell heights were higher than those derived from juvenile individuals for every intestinal segment evaluated (duodenum, ileum, and colon), which is consistent with a previous study that measured these parameters in adult and juvenile canine intestinal tissue samples [21]. Similar findings were described in a human sample population in 1988 when Thompson et al. reported shorter enterocytes in infants compared to healthy adult intestinal cells [22]. Furthermore, a recent human study described similar findings where infant organoids had a shorter cell height compared to adult intestinal enteroids [23]. These findings support the use of healthy adult canine intestinal samples, which can be obtained via endoscopy, for adult stem cell isolation and organoid generation. These results also suggest that adult canines, which are more readily available from established colonies or from clinical patient populations as compared to neonatal or juvenile canines, may be more optimal for organoid production and in vitro assays. Furthermore, these organoids can then be compared to histological samples obtained from the matching donor before and after in vivo treatment. In addition to potential use in 2D permeability assays, canine organoids may also serve as disease models (i.e., inflammatory bowel disease [24,25]) and drive personalized medicine as with human organoids (reviewed by Wang et al. [26]).

Cell and organoid histology provided insights into cell shape via measurements of cellular height and morphology, (i.e., mucin production as previously reported [12]). Representative organoids from each intestinal segment demonstrated luminal mucus. Goblet cell and enteroendocrine cell differentiation have been previously demonstrated in canine ENT cultures [10]. TEM also demonstrated apical vacuoles containing mucin-like material in duodenal organoid cells, consistent with the observation of mucin production by ENTs in this experiment. Cell polarization was determined by observing columnar morphology with basally located nuclei on light microscopy, and desmosomes and apical microvilli with basally located nuclei using TEM. Tight junctions, desmosomes, and apical microvilli were present in all four cultures; however, MDCK cells had subjectively fewer apical microvilli and wider intercellular spaces consistent with their polygonal to flattened morphology and tendency to pile.

To assess monolayer integrity in the 2D culture system, cell monolayers were grown for 13–14 days, with the timing determined based on published criteria for culture readiness [27,28,29]. MDCK cells on a permeable support system are considered ready for permeability assays as early as Day 3 to 4 [27,28] of culture, following an initial TEER spike and transition to a stationary growth phase [2]. Caco-2 monolayers are typically ready for permeability testing after reaching a plateau phase, with TEER values above 300 Ω·cm^2^ indicating acceptable monolayer integrity [29]. Canine ENTs (145 ± 54 Ω*cm^2^) closely approximated reported values for human small and large intestines (50–100 Ω*cm^2^ and 300–400 Ω*cm^2^, respectively) [15,16]. By comparison, the conventional cell lines (Caco-2 and MDCK) did not approximate TEER values reported for human small and large intestines. While these cell lines might not be expected to replicate small intestine, considering Caco-2 cells are derived from human colonic cells and MDCK cells are derived from canine kidney, Caco-2 cells grown in culture often exhibit properties of small intestinal epithelium [30,31]. Additionally, the TEER variance was higher in Caco-2 compared to MDCK and canine ENTs, suggesting that monolayer stability was lowest in the Caco-2 cells.

Overall, these findings support the use of canine intestinal organoid cultures in a 2D dual-chamber system when compared to conventional 2D cell cultures. However, functional assessments of these cultures are needed to validate and optimize their use in permeability assay evaluations.

### Study Limitations

While canine organoid cultures have potential advantages highlighted in this study, several limitations should be noted. The lack of standardization for canine intestinal organoid cultures in both 3D and 2D systems has been previously mentioned, and our group has made efforts to streamline standardized culture techniques, now published in two manuscripts [12,17]. Additionally, organoid culture upkeep is relatively expensive compared to conventional permeability assay cultures, which may result in scalability challenges when working with high volume, high throughput systems. This cost can be partially mitigated by using 2D L-WRN cell cultures that produce essential growth factors for the culture of organoids, thus eliminating the need for expensive recombinant growth factors such as Wnt-3a, R-spondin-3, and Noggin [32]. Despite this, limitations still exist with the use of Matrigel for organoid growth, as this animal-derived material varies naturally in composition which could lead to issues with reproducibility (reviewed by Penning and van den Boom [33]). A significant limitation of this study is the small number of organoid cell lines used in the experiments. While mucus vacuoles were observed in the duodenal organoid cells grown on 2D transwells, no mucus vacuoles were observed in the colon organoid cells grown on 2D transwells; this could be due to the fact that only a small subset of cells can be imaged at a time using TEM. The findings represent a preliminary effort to compare histological and semi-functional features (e.g., TEER) across cell lines used for intestinal permeability assessment, and further validation of this system is required. There are also some comparative considerations to account for in canine intestinal models, as dogs have Paneth-like cells, but lack distinct lysozyme-positive Paneth cells as are found in some species like mice and humans [10]. Additionally, this study does not include a comparison of P-gp expression, CYP450 expression, or apparent permeability estimates (Papp) between in vitro models and in vivo data, as this was beyond the scope of the current investigation. Future directions also include investigating techniques such as co-culture using pericryptal fibroblasts, which enhances cell differentiation [34], or immune cells. Further data supporting the use of canine organoid technology in veterinary drug development were published by our group elsewhere [35].

## 6. Conclusions

In conclusion, 3D canine ENT and COL were characterized via light microscopy, followed by culture in a 2D dual-chamber system to compare the biological relevance of various cell lines used in 2D biological model systems. The enterocytes of adult-stem cell-derived canine ENT and COL grown in a 2D monolayer maintained their columnar to tall cuboidal shape. Like Caco-2 and MDCK cells, they also formed tight junctions and desmosomes, which was supported by the duodenal ENT TEER measurements which more closely approximated the reported in vivo human intestinal values. Duodenal ENTs grown in 2D appear to have mucus production, which offers an advantage over Caco-2 and MDCK cells. The comparison between organoid 2D monolayers and 2D conventional cell cultures suggests the potential of the organoid model to be used in permeability assays in preclinical studies. Future steps include assessment of functional properties of the canine organoid monolayers, comparison to the in vivo results, and further repeated assessment of other cell lines used for in vitro intestinal modeling.

## Figures and Tables

**Table 1:** Canine intestinal organoids—measurement of enterocyte height.

	Adult Duodenum	Juvenile Duodenum	Adult Ileum	Juvenile Ileum	Adult Colon	Juvenile Colon	Caco-2	MDCK
Number of values	290	400	459	300	687	493	99	99
Median	14.2 ^d^	10.8 ^f^	17.4 ^c^	13.5 ^e^	19.7 ^a^	17.7 ^b^	13.8 ^de^	7.5 ^g^
Interquartile range (IQR)	6.2	3.6	5.9	7.2	6.1	5.4	6.6	2.6
Mean ranks	1149.0	582.1	1659.0	1078.0	2007.0	1715.0	1070.0	161.6

Table 1 compares the median height measurements of enterocytes in canine organoids derived from adult and juvenile duodenum, ileum, and colon, as well as Caco-2 and MDCK cells. Different letters between median cell heights in each group indicate statistical significance *p* < 0.001.

## Data Availability

The data are available upon request.

## Supplementary Materials

The following supporting information can be downloaded at https://www.mdpi.com/article/10.3390/biology14030270/s1. Figure S1: Median cell heights of juvenile and adult canine organoids grown in 3D culture as presented in Table 1. Error bars represent the interquartile range, and different letters between median cell heights in each group indicate statistical significance *p* < 0.001.
