# Supplementary material for: A Preclinical Model to Assess Intestinal Barrier Integrity Using Canine Enteroids and Colonoids
Source: Biology (Basel). 2025 Mar 6;14(3):270. doi: 10.3390/biology14030270 (PMC11939752; doi:10.3390/biology14030270)
Supplement: Supplementary file 1 [file biology-14-00270-s001.zip › biology-3492803-supplementary.pdf]

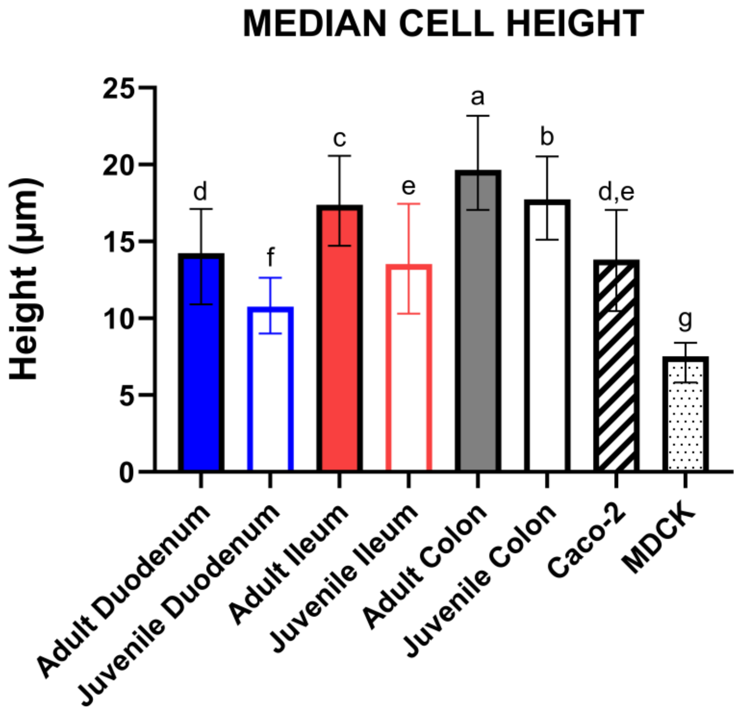

Figure S1. Median cell heights of juvenile and adult canine organoids grown in 3D culture as presented in Table 1. Error bars represent the interquartile range, and different letters between median cell heights in each group indicate statistical significance $p < 0.001$.

 https://doi.org/10.3390/xxxxx
